# Gene modification of the acetate biosynthesis pathway in *Escherichia coli* and implementation of the cell recycling technology to increase L-tryptophan production

**DOI:** 10.1371/journal.pone.0179240

**Published:** 2017-06-16

**Authors:** Qingyang Xu, Fang Bai, Ning Chen, Gang Bai

**Affiliations:** 1 State Key Laboratory of Medicinal Chemical Biology and College of Pharmacy, Tianjin Key Laboratory of Molecular Drug Research, Nankai University, Tianjin, China; 2 College of Biotechnology, Tianjin University of Science and Technology, Tianjin, China; Tsinghua University, CHINA

## Abstract

The implementation of a novel cell recycling technology based on a special disk centrifuge during microbial fermentation process can continuously separate the product and harmful intermediates, while maintaining the cell viability owing to the installed cooling system. Acetate accumulation is an often encountered problem in L-tryptophan fermentation by *Escherichia coli*. To extend our previous studies, the current study deleted the key genes underlying acetate biosynthesis to improve l-tryptophan production. The deletion of the phosphotransacetylase (*pta*)–acetate kinase (*ack*A) pathway in a *gltB* (encoding glutamate synthase) mutant of *E*. *coli* TRTHB, led to the highest production of l-tryptophan (47.18 g/L) and glucose conversion rate (17.83%), with a marked reduction in acetate accumulation (1.22 g/L). This strain, TRTHBPA, was then used to investigate the effects of the cell recycling process on L-tryptophan fermentation. Four different strategies were developed concerning two issues, the volume ratio of the concentrated cell solution and clear solution and the cell recycling period. With strategy I (concentrated cell solution: clear solution, 1: 1; cell recycling within 24–30 h), L-tryptophan production and the glucose conversion rate increased to 55.12 g/L and 19.75%, respectively, 17.55% and 10.77% higher than those without the cell recycling. In addition, the biomass increased by 13.52% and the fermentation period was shortened from 40 h to 32 h. These results indicated that the cell recycling technology significantly improved L-tryptophan production by *E*. *coli*.

## Introduction

Metabolic engineering is the purposeful modification of cellular activities aimed at improving the yield of a desired product through genetic engineering techniques [1]. l-Tryptophan, an essential amino acid for humans and animals, is widely used in the food, animal feed, and pharmaceutical industry, and it is therefore the subject of intensive research [2, 3]. Glutamate formation is a major obstacle in tryptophan production. Reduction in glutamate formation has been demonstrated to increase l-tryptophan production [4]. We previously studied the effect of gene modification of the glutamate synthesis pathway on l-tryptophan production and selected an *Escherichia coli* strain with mutated *gltB* (encoding glutamate synthase), designated as TRTHB, which was derived from the strain TRTH in our laboratory [2]. TRTH produces high levels of l-tryptophan and can achieve a high glucose conversion rate but exhibits high acetate accumulation [5, 6].

Acetate accumulation during l-tryptophan fermentation is also a major problem because of its inhibition of cell growth and L-tryptophan synthesis. L-tryptophan production has been shown to significantly improve by decreasing acetate accumulation [3, 7]. The conversion of acetyl-CoA through phosphotransacetylase (Pta) and acetate kinase (AckA) is the major pathway for acetate synthesis in *E*. *coli* [8], and the second pathway is the conversion of pyruvate directly into acetate via pyruvate oxidase B (*poxB*) [9]. The application of metabolic engineering to reduce carbon flow to the acetate biosynthesis pathway has been used to reduce the excretion of acetate in *E*. *coli* cultures [10], and the deletion of key genes and decrease in the activity of key enzymes of the acetate biosynthesis pathway are direct strategies to attenuate acetate accumulation [11]. The elimination of Pta, AckA, and PoxB activities has been found to result in a significant reduction in acetate accumulation [12]. The amino acid sequence of the recently identified *E*. *coli* propionate kinase (TdcD) exhibits high sequence identity with *E*. *coli* acetate kinase, and an *ackA*–*tdcD* mutant has been found to excrete lower concentrations of acetate compared with an *ackA* deletion mutant [7]. In previous studies, we deleted the *pta* gene of an *E*. *coli* TRTH derived L-tryptophan producing strain and the resulting strain accumulate lower concentrations of acetate and produce higher yields of l-tryptophan [3]; we deleted *ackA* and *tdcD* of TRTH, either separately or in combination, which all decreased acetate accumulation and increased L-tryptophan production, and the production of L-tryptophan and glucose conversion rate of strain TRTHAT (Δ*ackA*Δ*tdcD*) reached 52.5 g/L and 21.2%, respectively [7]. The effects of combinational deletion of *pta* and *ackA*/*tdcD*, and the deletion of *poxB* of TRTH derived strains on L-tryptophan production have yet to be investigated.

The implementation of integrated fermentation/membrane extraction process could reduce the inhibitory effects of products and intermediates such as acetate, to the product synthesis pathways and bacterial growth, meanwhile simplify the downstream product extraction process. However, the short service life of membrane due to concentration polarization and membrane fouling prohibits the industrial application of this technology. Recently, GEA Westfalia Separator Group in Germany developed a disk stack centrifugal separator with an online automatic sterilization system, which can be used to recycle the cells during fermentation while enables maintenance of cell viability owing to the cooling system installed. The inhibitory effect of acetate can be reduced by removing the accumulated acetate from a fermenter by applying the cell recycling technology [13]. Investigators are focusing on bioprocess engineering techniques that employ cell recycling because of its potential to produce greater yields of desired products [14]. When the rate of product formation is a positive linear function of cell concentration or is induced by an increase in cell volume during growth, the use of a cell recycling loop dramatically increases the rates and yields of product formation [15]. Moreover, the cell recycling process reduces production costs by shortening fermentation times [16]. For example, the technology of recycling enzymes and yeast cells overcomes the impediments to commercial production of cellulosic ethanol, such as the requirement of a high enzyme load and slow xylose fermentation, which increase ethanol production 2–3-fold [17]. However, not all bacteria are suitable for this purpose [16].

In the present study, we investigated l-tryptophan production using bacteria with deletions of *pta* and *ackA*/*tdcD*, and the deletion of *poxB*, responsible for the two acetate biosynthetic pathways, and analyzed the parental strain (TRTHB) and the mutants. Moreover, we adopted the cell recycling technology to increase the l-tryptophan production and glucose conversion efficiency.

## Materials and methods

### Bacterial strains, plasmids, and primers

All bacterial strains, plasmids, and primers are listed in Table 1.

**Table 1:** Strains, plasmids, and primers used in this study.

Name	Characteristics	Source
Strains		
TRTHB^a^	*trp*EDCBA+Tet^R^, Δ*tnaA*, Δ*gltB*	[6]
TRTHBPA	Derived from TRTHB, but Δ*pta* and Δ*ackA*	this study
TRTHBPT	Derived from TRTHB, but Δ*pta* and Δ*tdcD*	this study
TRTHBB	Derived from TRTHB, but Δ*poxB*	this study
TRTHBPAB	Derived from TRTHBPA, but Δ*poxB*	this study
Plasmids		
pKD46	Am^R^, λ Red-expressing vector	[18]
pKD3	Cm^R^, Template vector	[18]
pCP20	Am^R^, Cm^R^, FLP-expressing vector	[18]
Primers		
*pta*-P1	5′-GCTGGCGGTGCTGTTTTGTAACCCGCCAAATCGGCGGTAACGAAAGAGGATAAACCTTGAGCGATTGTGTAGGCTGGAG-3′^b^	this study
*pta*-P2	5′-TAGTGATTATTTCCGGTTCAGATATCCGCAGCGCAAAGCTGCGGATGATGACGAGATAACGGCTGACATGGGAATTAGC-3′^b^	this study
*pta*-P3	5′-GTTTCGGCAAATCTGGTTTCATC-3′	this study
*pta*-P4	5′-TGGTAAGTATGCAAAGTGGGATGG-3′	this study
*ackA*-P1	5’-CTGTCCCCGGCGAAACAAGCTAAAAAAATTAACAGAACGATTATCCGGCGTTGACATTGAGCGATTGTGT AGGCTGGAG-3’^b^	this study
*ackA*-P2	5’-CGGATCACGCCAAGGCTGACGCTGGTCAGACCGACGCTGGTTCCGGTAGGGATCAGTAACGGCTGACATGGGAATTAGC-3’^b^	this study
*ackA*-P3	5’-TGCCCAGCCACCACAATC-3’	this study
*ackA*-P4	5’-GTGGTAGTTTGCGACGAT-3’	this study
*tdcD*-P1	5’-GTGGGAGAGATCTCACTAAAAACTGGGGATACGCCTTAAATGGCGAAGAAACGGTTTGAGCGATTGTGTAGGCTGGAG-3’^b^	this study
*tdcD*-P2	5’-CATCCTGAACATCGTATACAAACTGTTTTAATCCGTAACTCAGGATGAGAAAAGAGTAACGGCTGACATGGGAATTAGC-3’^b^	this study
*tdcD*-P3	5’-CGGGCGGACCAAATGATAC-3’	this study
*tdcD*-P4	5’-AACCCGAACATCCTTGAC-3’	this study
*poxB*-P1	5'-CCTCCTTTCTCTCCCATCCCTTCCCCCTCCGTCAGATGAACTAAACTTGTTACCGTTTTGAGCGATTGTGTAGGCTGGAG-3’^b^	this study
*poxB*-P2	**5’-**GTATCACTGCGTAAATCAATCATGGCATGTCCTTATTATGACGGGAAATGCCACCCTAACGGCTGACATGGGAATTAGC-3’^b^	this study
*poxB*-P3	5'-TTGGTTCTCGCATAATCGCCTTA-3’	this study
*poxB*-P4	**5'-C-CGGAAAGCTCTGCTGCGTAGTC-3'**	this study

Notes:

^a.^ the strain TRTHB was stored at the Culture Collection of Tianjin University of Science and Technology (collection number TCCC27005)

^b.^ the underlines indicate 56-nt regions of similarity of a target knockout gene.

### Media

The media used for generating and propagating recombinant strains were prepared according to published procedures [3]. The seed medium contained the following components (in g/L): glucose, 20; yeast extract, 15; sodium citrate, 0.5; (NH_4_)_2_SO_4_, 10; MgSO_4_·7H_2_O, 5; KH_2_PO_4_, 1.5; FeSO_4_·7H_2_O, 0.015; and vitamin B1, 0.1. The fermentation medium for producing l-tryptophan contained the following components (in g/L): glucose, 20; yeast extract, 1; sodium citrate, 2; (NH_4_)_2_SO_4_, 4; MgSO_4_·7H_2_O, 2; KH_2_PO_4_, 2; and FeSO_4_·7H_2_O, 0.15. The pH of both seed and fermentation media was adjusted to 7.0 with 4 mol/L NaOH. When required, the antibiotics ampicillin, chloramphenicol, and tetracycline were added at final concentrations of 100, 30, and 50 μg/mL, respectively.

### Culture conditions

Culture conditions used for the construction of recombinant strains and plasmids were controlled according to published procedures [3]. Fermentations were carried out in a 30-L fermenter. A 500-mL baffled flask containing 30 mL seed medium was inoculated with a single colony strain generated in the present study and cultivated at 36°C with shaking at 200 rpm for 12 h. A 30-mL inoculum of this culture was added aseptically to a 5-L seed fermenter (Biotech-2002 Bioprocess Controller, Baoxing, Shanghai, China) containing 3 L seed medium and cultivated at 36°C for 16 h. The culture grown in the seed fermenter was inoculated aseptically (10% v/v) into 18 L of fermentation medium in a 30-L fermenter. The temperature was maintained at 36°C, and the pH was adjusted to 7.0 with 25% ammonium hydroxide (w/w) during the cultivation period, and the level of dissolved oxygen (DO) was controlled at 20% (0–20 h) and 30% (20–38 h). When the initial glucose was depleted, glucose solution (80% w/v) was added to the fermenter according to the DO feedback strategy [19].

### Construction of mutant *E*. *coli* strains

Single and multigene knockout mutants were constructed as described previously [3, 20]. The *pta* gene was disrupted using the λRed helper plasmid pKD46. The appropriate amplicons were generated using the primers *pta*-P1 and *pta*-P2 with the helper plasmid pKD3. To eliminate Cm^R^ from the integrated locus, the cells were transformed with plasmid pCP20 carrying the gene encoding FLP recombinase. All PCRs were performed using the primers *pta*-P3 and *pta*-P4. Disruption of *ackA*, *tdcD*, and *poxB* using the primer pairs *ackA*-P1-*ackA*-P2 and *ackA*-P3-*ackA*-P4, *tdcD*-P1-*tdcD*-P2 and *tdcD*-P3-*tdcD*-P4, and *poxB*-P1-*poxB*-P2 and *poxB*-P3-*poxB*-P4 was performed as performed for *pta* deletion. The colonies with deleted genes were selected by colony PCR, and gene knockout was further confirmed by sequencing the PCR products.

### Analysis of fermentation products

Biomass, cell count, and l-tryptophan concentration in the fermentation broth were determined as described previously [2]. The concentrations of glucose, glutamate, and lactate were monitored using an SBA-40C biosensor analyzer (Biology Institute of Shandong Academy of Sciences, Jinan, China). Acetate concentrations were measured using a Bioprofile 300A biochemical analyzer (Nova Biomedical, Waltham, MA, USA).

### Analysis of metabolic flux

On the basis of metabolic flux balance analysis and the stoichiometry model, the distribution of metabolic flux with different strains during l-tryptophan production was calculated by MATLAB [6, 21].

### Presentation of the cell recycling strategy

**Fig 1:**
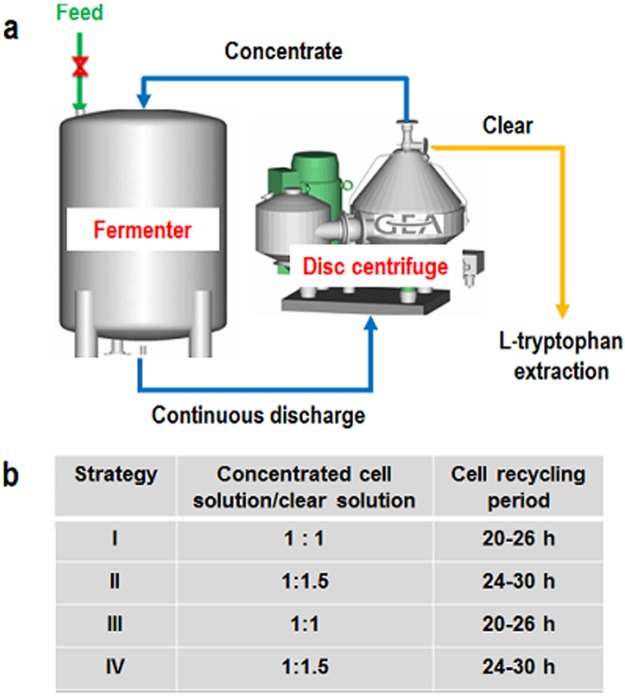
Schematic diagram illustrating the workflow of the cell recycling bioreactor and four cell recycling strategies adopted.

The cell recycling strategy to produce l-tryptophan was performed by combining a fermenter with a disk stack centrifugal separator equipped with an online automatic sterilization system (GEA Westfalia Separator HSD 1-06-107; Oelde, Germany). The schematic diagram illustrating the workflow of this cell recycling bioreactor is shown in Fig 1. During fermentation, the broth continuously entered the disk stack centrifugal separator; the clear solution discharged from the centrifuge was extracted to isolate l-tryptophan, and the concentrated cells were returned to the fermenter. The flow rate of the clear solution was maintained according to the feeding rate of the glucose solution to maintain the volume balance. Four cell recycling strategies were developed: strategy I, 1:1 (v/v) concentrated cell solution: clear solution, cell recycling within 20–26 h of fermentation; strategy II, 1:1 (v/v) concentrated cell solution: clear solution, cell recycling within 24–30 h; strategy III, 1:1.5 (v/v) concentrated cell solution: clear solution, cell recycling within 20–26 h; and strategy IV, 1:1.5 (v/v) concentrated cell solution: clear solution, cell recycling within 24–30 h (Fig 1).

### Statistical analysis

All experiments were conducted in triplicate, and the data were averaged and presented as the mean ± standard deviation. One-way analysis of variance followed by Dunnett’s multiple comparison test were used to determine significant differences. Statistical significance was defined as *p* < 0.05.

## Results

### Process analysis of L-tryptophan production and construction of recombinant *E*. *coli*

#### Process analysis of L-tryptophan production by the strain TRTHB

**Fig 2:**
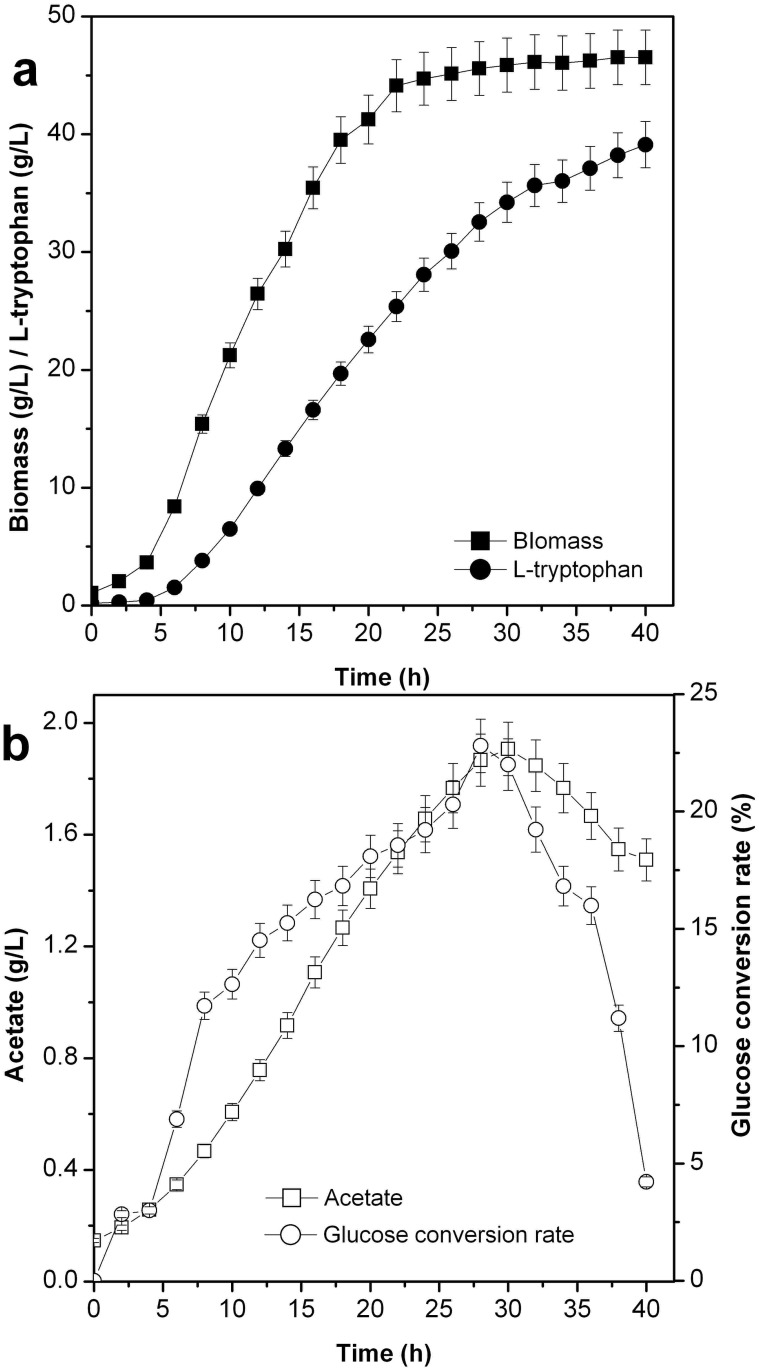
Analysis of process parameters in L-tryptophan production by using the strain TRTHB (*P*<0.05).

The process parameters of l-tryptophan production using the strain TRTHB are displayed in Fig 2. The biomass yield and l-tryptophan production were 46.53 g/L and 39.12 g/L, respectively. In l-tryptophan fermentation, acetate accumulation occurred, and the acetate was consumed during the later fermentation period (26–40 h). The accumulation of acetate was 1.51 g/L at the end of the fermentation. Glucose as the carbon source was linked to tryptophan, and the total glucose conversion rate was 14.89%.

#### Construction of recombinant *E*. *coli* with mutations in acetate biosynthesis genes

The genes *pta*, *ackA*, *tdcD*, and *poxB* are the key genes for acetate synthesis. These four genes were deleted to construct mutants to reduce acetate accumulation. The deletions of *pta*, *ackA*, *tdcD*, and *poxB* were confirmed using PCR with the primer pairs *pta*-P3-*pta*-P4, *ackA*-P3-*ackA*-P4, *tdcD*-P3-*tdcD*-P4, and *poxB*-P3-*poxB*-p4, respectively. The lengths of the amplified DNA fragments and the sequences of PCR products indicated that the deletions of *pta*, *ackA*, *tdcD*, and *poxB* were successful.

### L-Tryptophan production by the mutant strains

#### Biomass yield and L-tryptophan production

**Fig 3:**
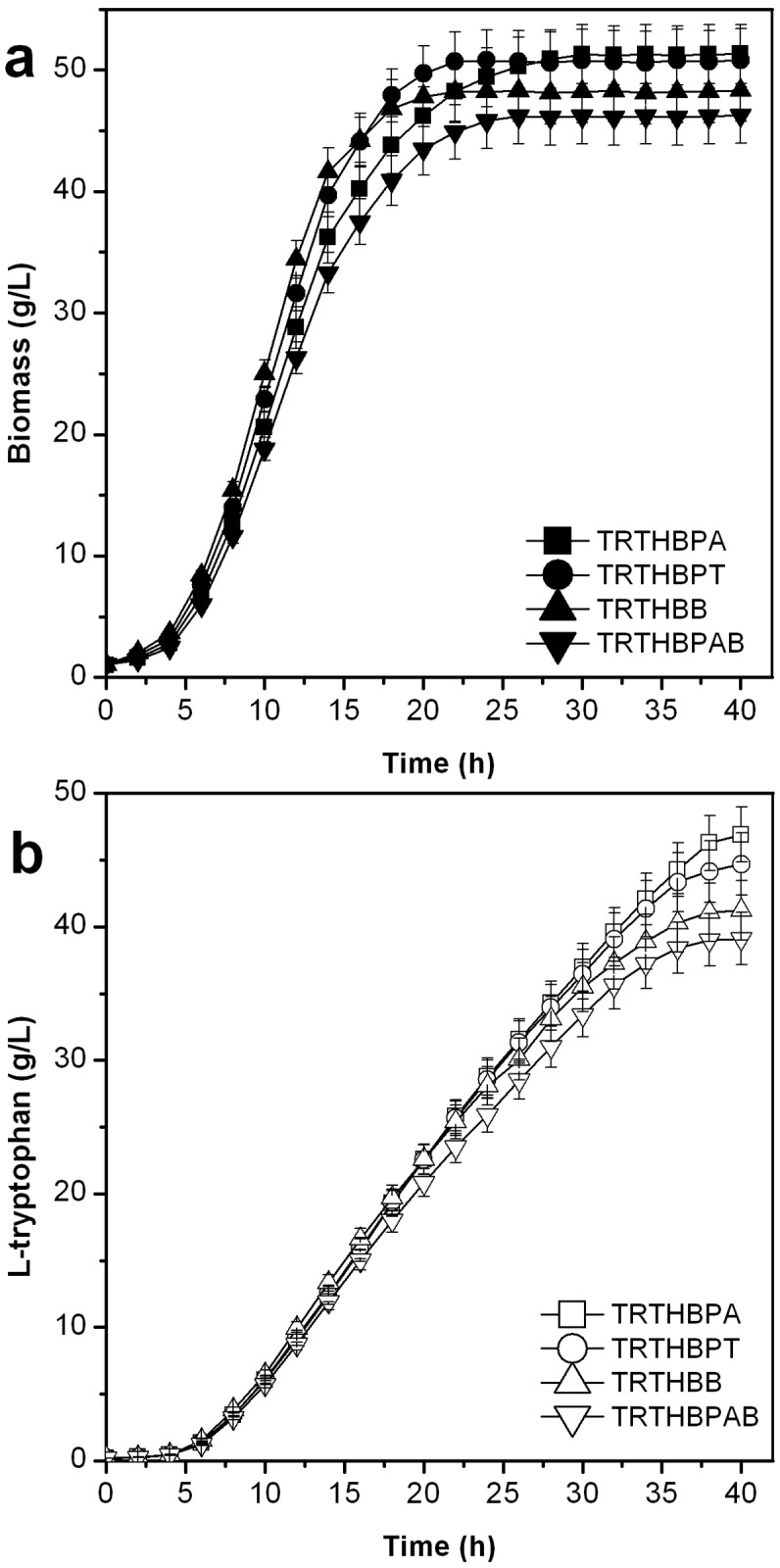
Effect of modifying the genes required for acetate synthesis on biomass and production of L-tryptophan in L-tryptophan fermentation (*P*<0.05).

The biomass yield and l-tryptophan production of the strains with acetate synthesis gene modifications are presented in Fig 3. The biomass yield and l-tryptophan production by TRTHB were higher than those by the mutants during the early fermentation period; however, at the end of the fermentation, the values of the mutants were higher than those of TRTHB. The biomass yield (51.87 g/L) and l-tryptophan production (47.18 g/L) obtained with THTHBPA were the highest, 11.48% and 20.60% higher, respectively, than those of TRTHB. The biomass yield and l-tryptophan production of strains TRTHBPA and TRTHBPT were higher than those of TRTHBB. TRTHBPAB had the lowest biomass yield (47.27 g/L) and l-tryptophan production (39.61 g/L) among all mutants.

#### Acetate accumulation and glucose conversion rate

**Fig 4:**
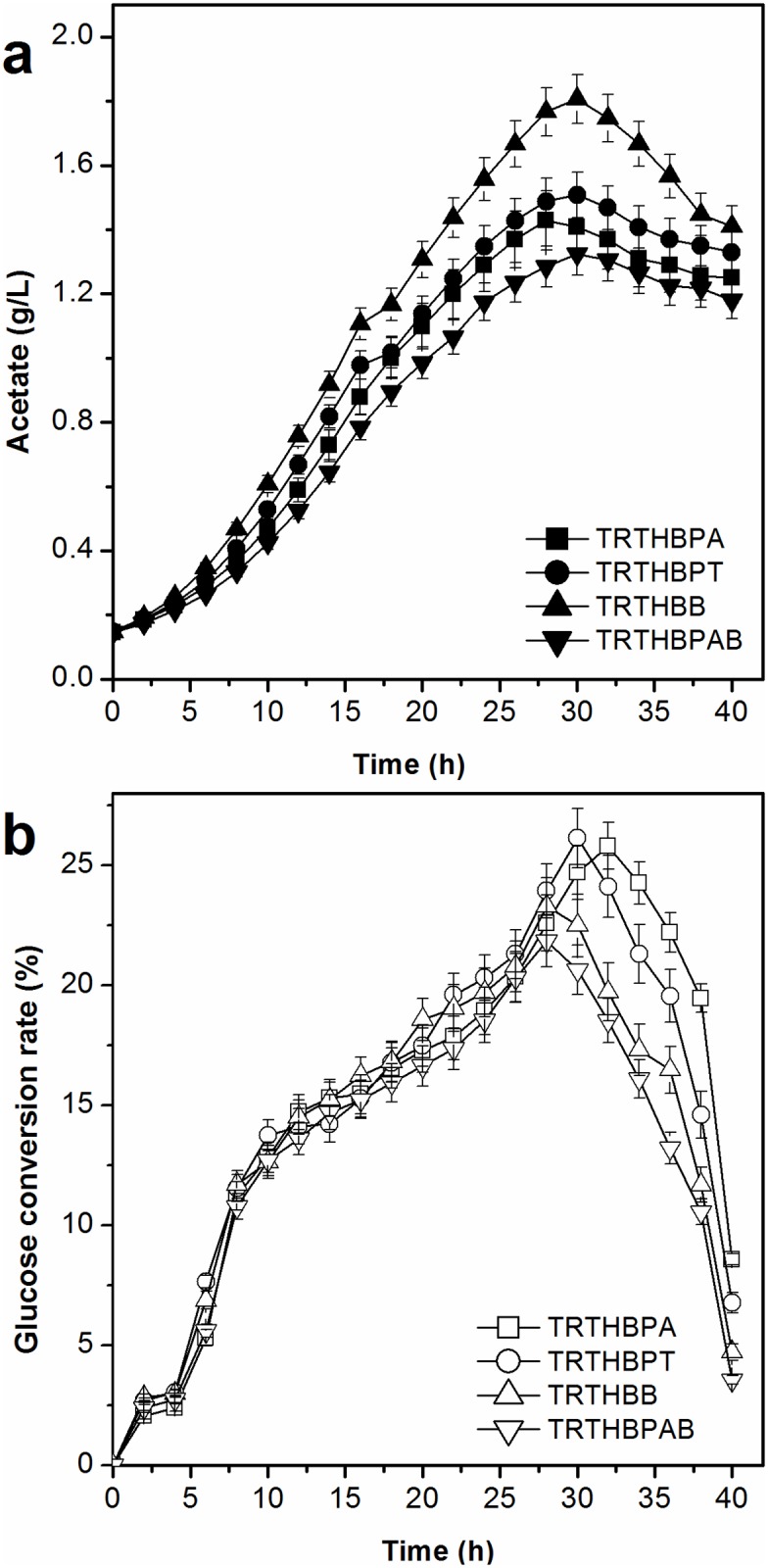
Effect of modifying the genes required for acetate synthesis on accumulation of acetate and glucose conversion rate in L-tryptophan fermentation (*P*<0.05).

Acetate accumulation and glucose conversion rate by the mutant strains are presented in Fig 4. Acetate levels accumulated by the mutants lacking the genes for acetate synthesis were lower than those by TRTHB. Acetate levels accumulated by TRTHBPA (1.25 g/L) and TRTHBPT (1.33 g/L) were lower than those accumulated by TRTHBB (1.41 g/L). TRTHBPAB produced the lowest concentration of acetate (1.18 g/L), 21.85% lower than that by TRTHB (1.51 g/L).

The glucose conversion rate of glucose by TRTHBPAB was the lowest throughout the fermentation phase. The glucose conversion rate by TRTHBB was lower than those by TRTHBPA and TRTHBPT during the later fermentation period. The glucose conversion rates by TRTHBPA, TRTHBPT, TRTHBB, and TRTHBPAB were 17.83%, 17.42%, 16.05%, and 15.67%, respectively, which were higher than that by TRTHB (14.89%).

#### Distribution of metabolic flux

**Fig 5:**
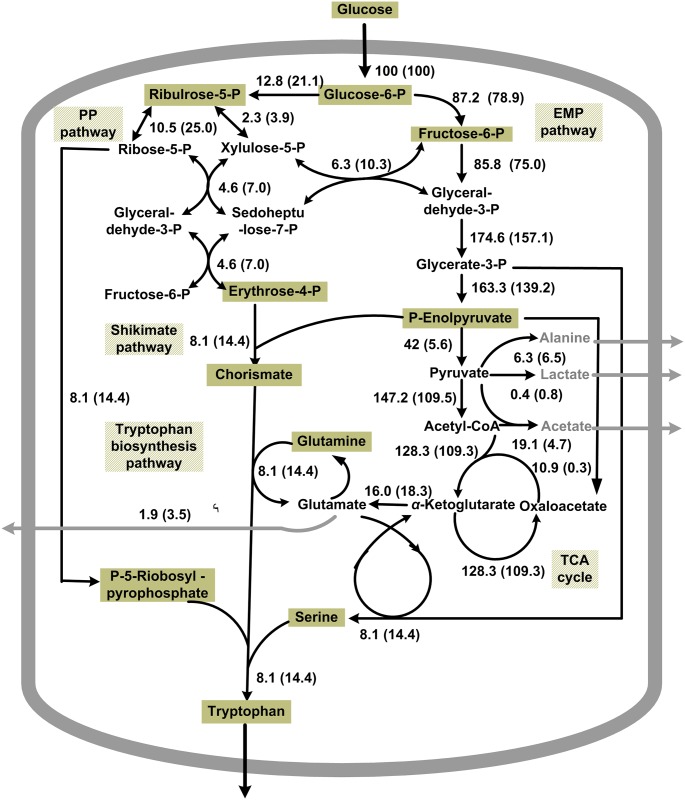
The metabolic flux distribution of TRTHB and TRTHBPA during the later fermentation period (30–40 h) of L-tryptophan production. Values in parentheses represent the metabolic flux of TRTHBPA.

On the basis of the above results, the strain TRTHBPA was found to be the best strain for producing l-tryptophan among the strains studied. The metabolic flux distribution of TRTHB and TRTHBPA during the later fermentation period (30–40 h) of l-tryptophan production is presented in Fig 5. Compared with TRTHB, the metabolic flux that entered the EMP pathway decreased by 13.39%, and that entering the pentose phosphate (PP) pathway increased by 80.47% in TRTHBPA. In TRTHBPA, the acetate flux decreased from 19.1% to 5.1%, and the flux for glutamate and lactate increased. Compared to TRTHB, in TRTHBPA, more phosphoenolpyruvate and erythrose-4-phosphate entered the shikimate pathway. This increased the metabolic flux of tryptophan to 15.1%, which was 1.86 times higher than that of TRTHB.

### Application of the cell recycling technology

The mutants lacking the genes of the acetate biosynthesis pathway demonstrated decreased acetate accumulation and increased l-tryptophan production; however, acetate was also excreted during l-tryptophan production. Meanwhile, the rate of l-tryptophan formation is a positive linear function of cell concentration. The cell recycling technology was applied in l-tryptophan production using TRTHBPA to improve the cell concentration and thereby increase the rates and yields of l-tryptophan formation, and to shorten the fermentation period at the same time. Based on the analysis of cell proliferation and L-tryptophan synthesis during the fermentation process, two main issues were considered: the volume ratio of concentrated cell solution and clear solution and the cell recycling period. When the lower ratio of concentrated and clear solution was maintained, the stronger centrifugal strength was needed, which influenced the cell activity due to the higher shear force, although the higher concentrations of acetate and L-tryptophan were removed. At the early stage of fermentation, the cell density and L-tryptophan concentration were low and the cell recycling was not beneficial for L-tryptophan production; after 20 h of fermentation, the biomass almost reached the highest value and L-tryptophan began to crystallize (at about 20 g/L) and the cell recycling could enhance L-tryptophan production (Fig 3). Therefore, four different strategies were developed as stated in the materials and methods section (Fig 1).

#### Biomass and production of L-tryptophan

**Fig 6:**
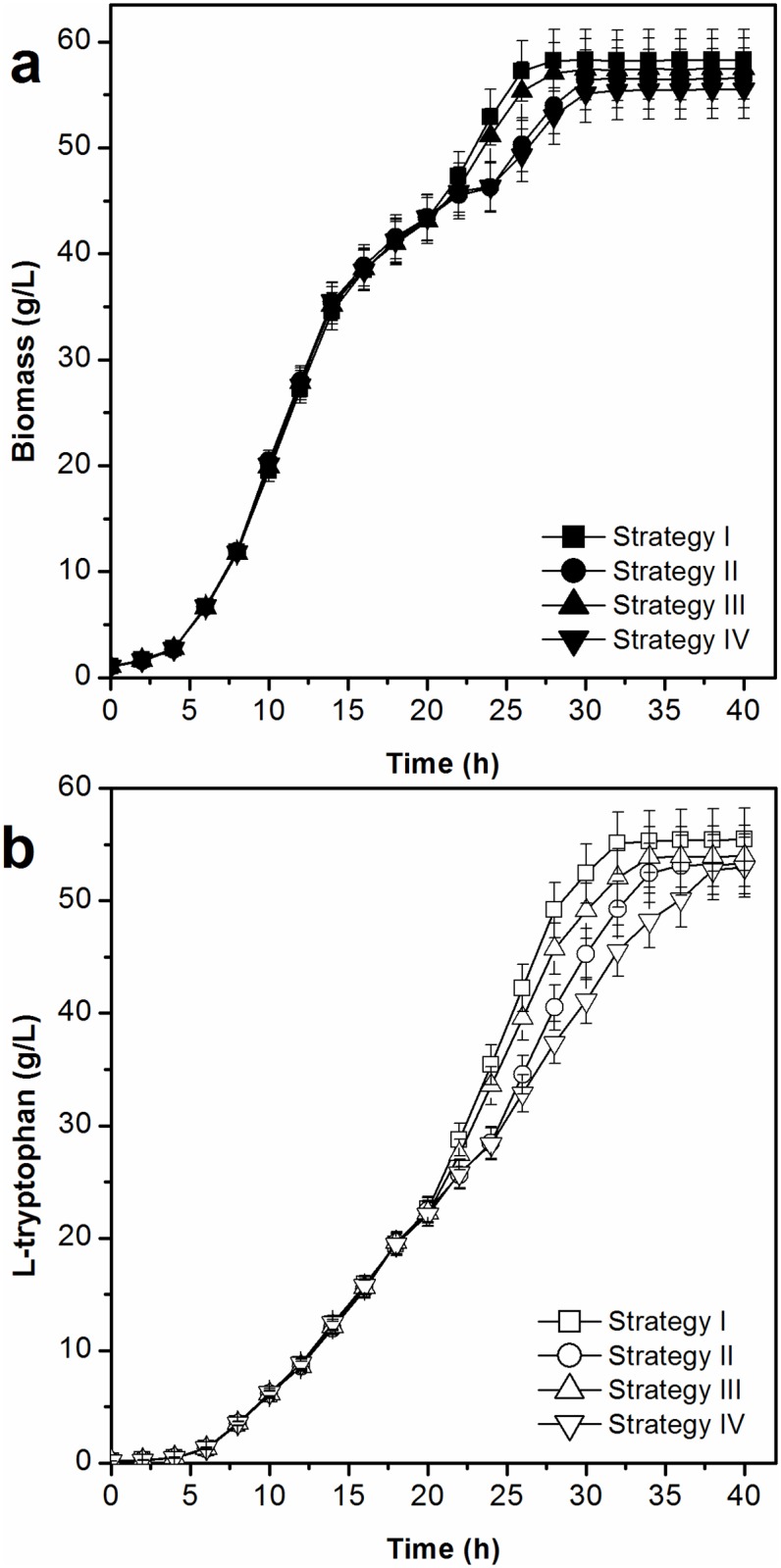
Effect of a cell recycle strategy on biomass and production of L-tryptophan in L-tryptophan fermentation by TRTHBPA (*P*<0.05).

**Fig 7:**
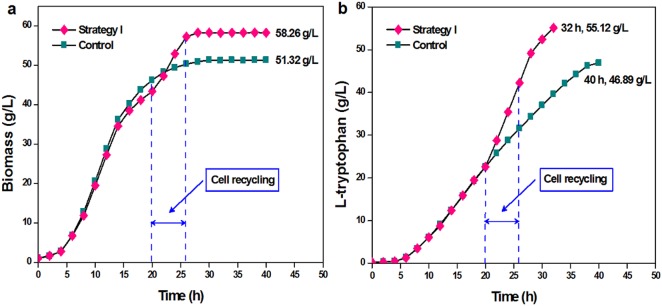
Comparisons of biomass (a), L-tryptophan production and fermentation period (b) in L-tryptophan fermentation by TRTHBPA between strategy I and the control (without cell recycling).

Fig 6 shows the biomass yields and l-tryptophan production levels using different cell recycling strategies. The application of cell recycling increased biomass yields and l-tryptophan production. The highest biomass yield (58.26 g/L) was obtained using cell recycling strategy I, and the biomass yield using cell recycling strategies II, III, and IV were 56.57 g/L, 57.45 g/L, and 55.52 g/L, respectively. Accordingly, the time to produce l-tryptophan using cell recycling strategies I, II, III, and IV were shortened from 40 h without cell recycling to 32 h, 36 h, 34 h, and 38 h, respectively. Using cell recycling strategies I, II, III, and IV, the concentrations of l-tryptophan were 55.12 g/L at 32 h, 53.15 g/L at 36 h, 53.87 g/L at 34 h, and 52.72 g/L at 38 h, respectively. The comparisons of biomass, L-tryptophan production and fermentation period between strategy I and the control (without cell recycling) are presented in Fig 7.

#### Acetate accumulation and glucose conversion rate

**Fig 8:**
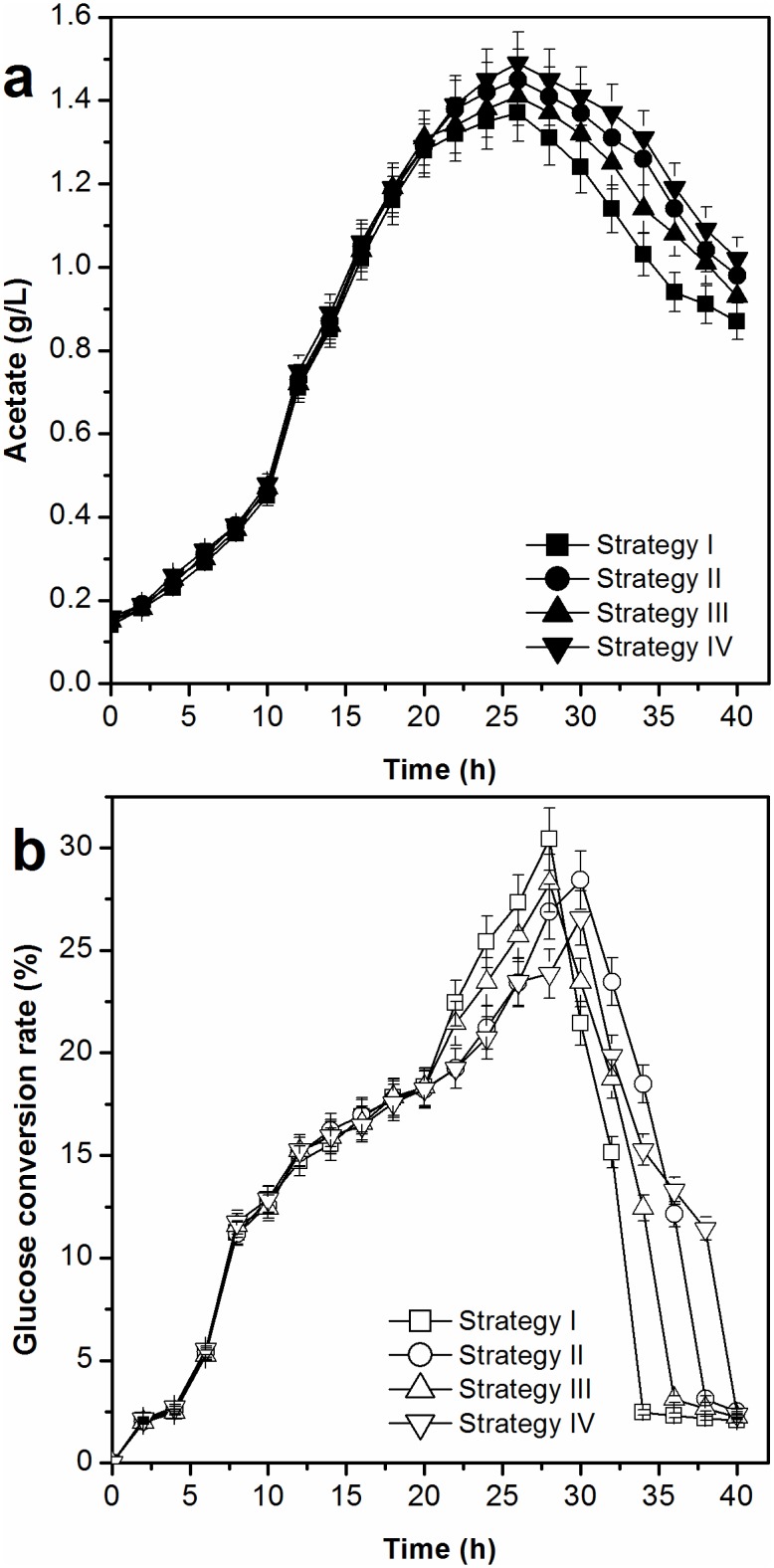
Effect of a cell recycle strategy on accumulation of acetate and glucose conversion rate of L-tryptophan in L-tryptophan fermentation by TRTHBPA (*P*<0.05).

The acetate accumulation levels and glucose conversion rates using the cell recycling strategies are displayed in Fig 8. Acetate accumulation was reduced by applying the cell recycling strategy. Acetate accumulation with cell recycling strategy I was the lowest (0.87 g/L), whereas the corresponding values for cell recycling strategies II, III, and IV were 0.98 g/L, 0.93 g/L, and 1.02 g/L, respectively. The glucose conversion rate was higher during the cell recycling period, thereby increasing the total glucose conversion rate. The total glucose conversion rates using cell recycling strategies I, II, III, and IV were 19.75%, 19.14%, 19.35%, and 18.87%, which were higher than that without cell recycling (17.83%).

#### Relation between cell biomass and tryptophan biosynthesis

**Fig 9:**
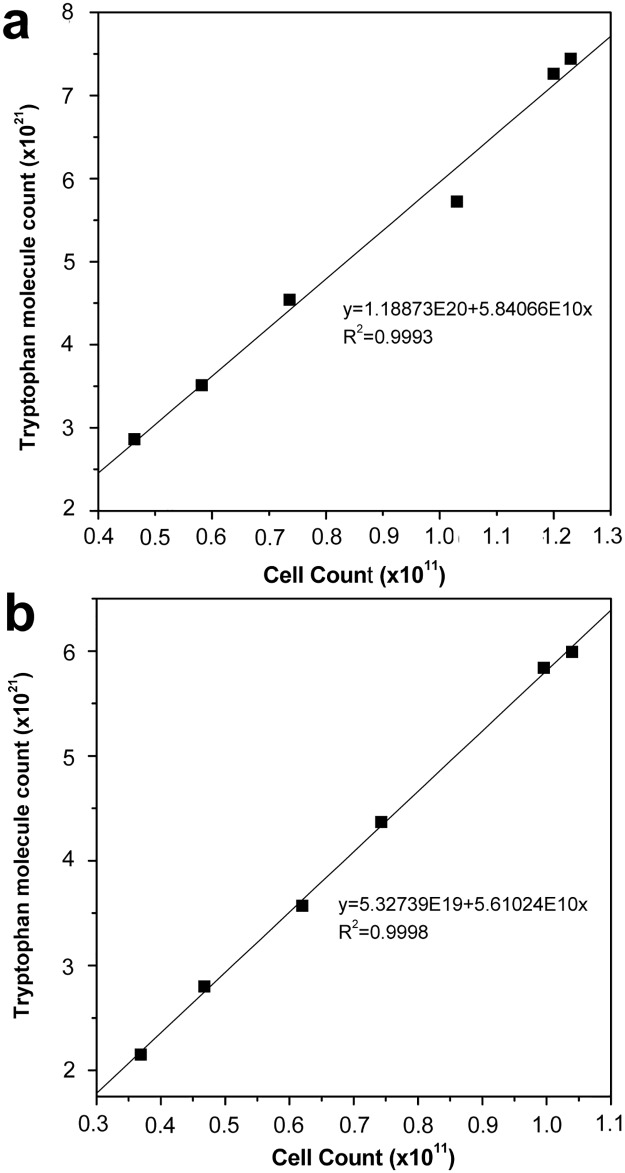
The linear relation of cell and tryptophan biosynthesis during cell recycling period (20–26 h) with strategy I and III.

According to the biomass yield and l-tryptophan production in cell recycling strategies I and III (Fig 5) and no cell recycling (Fig 2), the linear relation of cell biomass and tryptophan formation with strategy I and III during the fermentation period (20–26 h) was determined (Fig 9). The linear equations of cell increment (x) and tryptophan molecules (y) with strategy I and III were y = 1.18873E20 + 5.84066E10x (R^2^ = 0.9993) and y = 5.32739E19 + 5.61024E10x (R^2^ = 0.9998), respectively. During the cell recycling period (20–26 h), 0.097 pmol l-tryptophan was generated by an increase in one cell when using strategy I, which was 4.11% higher than that when using strategy III.

## Discussion

### Process analysis of L-tryptophan production and construction of recombinant *E*. *coli*

The acetate excreted in l-tryptophan production by the strain TRTHB inhibits cell growth and l-tryptophan formation, and biomass yield and l-tryptophan production increase with the reduction in acetate accumulation [2, 3]. The genes *pta*, *ackA*, and *poxB* encode the key enzymes of acetate synthesis; therefore, the decrease in or elimination of the activity of these key enzymes reduces the excretion of acetate [21, 22]. TdcD is a propionate/acetate kinase and plays an important role in the formation of acetate [18], and *tdcD* exhibits a high identity with *ackA* from TRTHB [23]. We therefore selected the four genes *pta*, *ackA*, *tdcD*, and *poxB* to be deleted to construct mutants accumulating low concentrations of acetate.

### L-Tryptophan production by the mutant strains

The deletion of certain genes changes the distribution of the metabolic flux within a cell. For example, the growth rate of an *E*. *coli pta* mutant perturbs the flux of acetyl-CoA [24]. Moreover, mutants with deletions of the *ackA*–*tdcD* genes grow more slowly than *ack*A or *tdc*D single mutants do [7]. The biomass of TRTHB is higher than that of the mutants during the initial fermentation period. The metabolic flux of acetate synthesis is decreased in mutants with deletions of the genes required for acetate synthesis, and the reduced acetate flux is redirected to the excretion of pyruvate, lactate, and glutamate [4, 7]. In the present study, the mutants accumulated lower concentrations of acetate than those of the parental strain TRTHB. The inhibition of cell growth and l-tryptophan formation caused by acetate can be reversed by reduced acetate concentrations, which then leads to increases in biomass yields and l-tryptophan production [6, 7]. We found that the l-tryptophan production and glucose conversion rate of the mutants with deletions of acetate synthesis genes were higher than those of TRTHB. The lowest concentration of acetate accumulated in cultures of TRTHBPAB, although its biomass and l-tryptophan production were lower than those of other mutants, which might be ascribed to the effect of the deletion of multiple genes related to cell growth and tryptophan formation [2, 3].

Because *pta*–*ackA* and *pta*–*tdcD* are involved in the primary pathway for acetate synthesis, the concentration of acetate accumulating in cells with mutations in these genes has been found to be lower than that of the *poxB* mutant [10, 19]. Meanwhile, the deletion of *pta*–*ackA* in TRTHB led to the excretion of lower concentrations of acetate than the mutant with a disruption of *pta*–*tdcD*, which implied that *ackA* was more important for acetate kinase activity [7, 18]. The strain TRTHBPA with *pta*–*ackA* deletions produced the highest L-tryptophan (47.18 g/L) and glucose conversion rate (17.83%), which were lower than the corresponding values of TRTHAT with *ackA*–*tdcD* deletions in our previous study [7], implying the combinational deletions of *pta*–*ackA/tdcD* was not an optimized strategy. Nevertheless, TRTHBPA constructed in this study was chosen for investigating the effects of the cell recycling operation.

Numerous studies show that in cells lacking acetate synthesis genes, the carbon flux through the EMP pathway decreases, whereas the carbon flux through PP increases, which increases tryptophan synthesis [1, 3]. Because of the reduced accumulation of acetate and the increased metabolic flux towards tryptophan biosynthesis, the strain TRTHPA produced the highest yields of l-tryptophan, and TRTHBPA had the highest glucose conversion rate (17.83%, 1.21 times higher than that of TRTHB). Owing to the enhancement flux of the shikimate pathway, the tryptophan biosynthesis flux of TRTHBPA was 86.42% higher than that of TRTHB. Despite the deletion of key genes for acetate synthesis, acetate still accumulated in l-tryptophan production. Acetate accumulation is mediated by the combination of high rates of glucose uptake and catabolism through the EMP pathway, which increases the rate of Ac-CoA synthesis and, consequently, surpasses the capacity of the tricarboxylic acid cycle to completely consume acetate [24]. Most processes design or introduce genetic modifications to overcome acetate formation with the ultimate aim to bring growth rate and oxygen consumption into a better balance [13].

### Cell recycling technology

Application of a cell recycling strategy increases the production of the desired product, as well as its production capacity, making it an attractive technology for the production of a variety of bacterial products [15]. In the bioconversion of lignocellulosic sugars to ethanol, the application of the cell recycling technology reduces capital costs, processing time, and biocatalyst costs, which are achieved by reducing bioprocessing time, increasing biofuel productivity, and recycling biocatalysts [16]. Cell recycling technologies increase cell density, and the clear fluid that is removed from the fermenter decreases the concentration of acetate, leading to decreased inhibition of l-tryptophan formation and an increase in l-tryptophan production [6, 7].

We show here that the concentration of acetate at 1:1 v/v (concentrated cell solution: clear solution) was lower than that at 1:1.5 v/v (concentrated cell solution: clear solution). During the cell recycling process, cells grow and die [25], and the biosynthetic activity of a cell decreases with increased shear-force during centrifugation [15]. In the present study, cell metabolism at 1:1 v/v (concentrated cell solution: clear solution) was higher than that at 1:1.5 v/v (concentrated cell solution: clear solution). The l-tryptophan generation capacity of cells with strategy I was 4.11% higher than that with strategy III. The cell recycling process increased the rate of l-tryptophan production and shortened fermentation time. The biomass yield and l-tryptophan production obtained with strategy I were 12.32% and 16.53% higher, respectively, than those without cell recycling [13]. Furthermore, owing to the high levels of metabolic activity and biomass, as well as the low concentration of acetate, the highest conversion efficiency of glucose obtained increased by 10.77%, and the fermentation period was shortened by 20% with strategy I compared to the absence of cell recycling [14].

## Conclusion

In the present study, we found that acetate accumulated in l-tryptophan production using TRTHB, and the mutants of key genes (*pta*, *ackA*, *tdcD*, and *poxB*) of acetate synthesis accumulated low concentrations of acetate and yielded high levels of l-tryptophan. The deletion of *pta*–*ackA* in THTRB produced the highest titers of l-tryptophan. Meanwhile, the cell recycling technology was applied in l-tryptophan production using the strain TRTHBPA, and l-tryptophan production and glucose conversion efficiency improved markedly because of the higher cell concentration. The tryptophan formation capacity per cell increased with the application of an appropriate cell recycling strategy. Under the optimized cell recycling strategy, the biomass and L-tryptophan production increased by 13.52% and 17.55% and the fermentation period decreased by 20%, respectively, compared to those without the cell recycling.
